# Critical periods of increased fetal vulnerability to a maternal high fat diet

**DOI:** 10.1186/1477-7827-12-80

**Published:** 2014-08-18

**Authors:** Maria del Mar Plata, Lyda Williams, Yoshinori Seki, Kirsten Hartil, Harpreet Kaur, Chia-Lei Lin, Ariana Fiallo, Alan S Glenn, Ellen B Katz, Mamta Fuloria, Maureen J Charron, Patricia M Vuguin

**Affiliations:** Department of Pediatrics, Albert Einstein College of Medicine, 10461 Bronx, NY USA; Department of Biochemistry, Albert Einstein College of Medicine, 10461 Bronx, NY USA; Department of Medicine, Albert Einstein College of Medicine, 10461 Bronx, NY USA; Department of Obstetrics and Gynecology and Women’s Health, Albert Einstein College of Medicine, 10461 Bronx, NY USA; Cohen Children’s Medical Center, Hofstra School of Medicine, 1991 Marcus Ave, 11402 Lake Success, NY USA

## Abstract

**Background:**

Fetal adaptations to high fat (HF) diet in utero (IU) that may predispose to Metabolic Syndrome (MetS) in adulthood include changes in fetal hepatic gene expression. Studies were performed to determine whether maternal exposure to HF diet at different stages during pregnancy had different effects on the fetus, including hepatic gene expression.

**Methods:**

Female wild type mice were fed either a HF or breeding chow (C) for 2 wks prior to mating. The experimental groups were composed of embryonic day (e) 18.5 fetuses obtained from WT female mice that were fed HF (HF, 35.5% fat) or breeding chow (C, 9.5% fat) for 2 wk before mating until e9.5 of pregnancy (periconception-midpregnancy). At e9.5 dams were switched to the opposite diet (C-HF or HF-C).

**Results:**

Exposure to HF diet throughout pregnancy reduced maternal weight gain compared to C diet (p < 0.02 HF vs. C). HF-C dams had significantly decreased adiponectin levels and litter size when compared to C-HF (p < 0.02 HF-C vs C-HF). Independent of the timing of exposure to HF, fetal weight and length were significantly decreased when compared to C diet (HF, C-HF and HF-C vs. C p < 0.02). HF diet during the second half of pregnancy increased expression of genes in the fetal liver associated with fetal growth (C-HF vs C p < 0.001), glucose production (C-HF vs C p < 0.04), oxidative stress and inflammation (C-HF vs C p < 0.01) compared to C diet.

**Conclusions:**

This model defines that there are critical periods during gestation in which the fetus is actively shaped by the environment. Early exposure to a HF diet determines litter size while exposure to HF during the second half of pregnancy leads to dysregulation of expression of key genes responsible for fetal growth, hepatic glucose production and oxidative stress. These findings underscore the importance of future studies designed to clarify how these critical periods may influence future risk of developing MetS later in life.

## Background

Epidemiological as well as animal data has demonstrated that fetal and early postnatal periods are important for determining the future risk of developing Metabolic Syndrome (MetS), regardless of genetic or additional environmental exposures. The process by which “insults at critical stages of development” lead to permanent changes in tissue structure or function that adversely affect physiological functions in adult life is known as the Developmental Origins of Health and Disease (DOHaD) [1, 2].

Pregnancy and lactation are critical periods where maternal diet plays an important role in fetal development and the development of MetS [2–4]. Poor dietary choices during pregnancy are more likely to be encountered in groups with less education and lower socioeconomic status (SES) [5–7].

A Western Style diet, mostly high fat diet (HF) during pregnancy (in utero, IU) is associated with low, normal as well as increase birth weight [8–11]. In both humans and animals, many studies investigating the effects of HF in utero are complicated by the occurrence of confounding variables such as maternal obesity or gestational diabetes that also influence birth weight or the development of MetS in the offspring [12–14].

Fetal growth involves a carefully orchestrated cascade by which each organ develops throughout gestation. Insulin, glucocorticoids, Insulin-like Growth Factors (IGFs) and their regulatory binding proteins (IGFBPs) play an important role in fetal growth and development, which are regulated by maternal nutrient availability and maternal metabolic milieu [15, 16]. Poor prenatal growth, as seen in babies born small for gestational age (SGA), is associated with an increase in inflammation and oxidative stress at birth and insulin resistance in prepubertal children [17, 18] and is known to increase the risk of developing cardiovascular disease and impaired glucose tolerance in adulthood [19, 20].

Poor prenatal growth, an inflammatory-oxidative stress response, lipotoxicity in the liver and dysregulation in the hepatic expression of genes involved in gluconeogenesis (GNG) have also been observed in non-human primates as well as rodent models exposed to HF IU [9, 10, 21, 22]. Similarly, our laboratory has previously reported that WT mice exposed to HF IU are born small, have altered pancreatic islet morphology [23] and develop features of MetS compared to mice exposed to control (C) diet IU and during lactation [9]. Fetal liver of mice exposed to HF IU have increased expression of genes involved in GNG, inflammation and oxidative stress [10] that were associated with functional alterations to fetal hepatic histone modifications [24]. These modifications persisted up to 5 weeks of age [24], suggesting that prenatal exposures by themselves will lead to permanent adaptations with lasting effects on metabolic mechanisms.

The timing of the insult has the potential to critically influence the development and function of any particular organ. For example, it is known that during placental development, disruption during a period of angiogenesis will have different consequences compared to disruption during a period of growth and differentiation [25].

The aim of this study was to investigate the changes that occur in the fetus, particularly in the expression of genes in the fetal liver that are involved in fetal growth, hepatic glucose production, oxidative stress and inflammation, after exposure to a HF diet during specific periods of gestation. Defining the periods of increased vulnerability during fetal development to environmental programming will reveal windows of opportunity in which treatments may have increased likelihood for a positive outcome in the offspring. This is important as women of lower SES, at the greatest risk of malnutrition, do not access health care until they are already pregnant [26].

## Methods

Animals and Experimental Design: All studies were approved by the Institutional Animal Care and Use Committee at Albert Einstein College of Medicine, in accordance with Animal Welfare Act guidelines. Twelve to 14 week old wild-type (WT) female mice (CD1 background) were maintained on control diet (C; Pico Lab® Mouse Diet \#5058; 9% fat), or switched to high fat (HF; Bio-Serv Product #F3282; 35.5% fat as lard) diet during the periconceptional period, 2 weeks prior to mating. Females were bred to non-littermate glucose transporter 4 heterozygous (G4+/−) males, a model of insulin resistance [27]. In contrast to WT male offspring from WT pregnancies, WT male offspring of WT female and G4+/− male exposed to a maternal HF IU are programmed to develop characteristics of the MetS [9, 10, 23].

**Figure 1:**
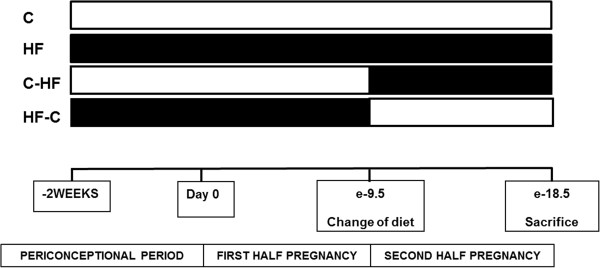
**Study design and groups based on maternal diet.** Control (C), High Fat (HF), Control – High Fat (C-HF) and High Fat – Control (HF-C); N = 5-10/group diet.

Pregnancy was determined by the presence of a copulatory plug and defined as embryonic day 0.5 (e0.5). Four experimental groups were studied (Figure 1). Pregnant females were kept on the same diet throughout periconception and pregnancy (C: n = 10; HF: n = 8) or, to identify the critical period in which exposure to HF diet may affect fetal growth or development, dams were switched at mid-pregnancy (embryonic day, e9.5) to the opposite diet (C-HF: n = 5; HF-C: n = 8). Mid pregnancy was chosen because full expression of key enzymes and transcription factors involved in insulin signaling are present from mid pregnancy or embryonic day 9.5 onward [28].

Maternal Phenotypic Evaluation: Body weight gain [(BW day X - BW e0.5)/BW day mating e18.5] x 100)] in pregnant females was determined (C: n = 10; HF: n = 8; C-HF: n = 5; HF-C: n = 8) as previously described [9]. Metabolic phenotype of each mother was determined at the time of sacrifice (Table 1). Blood glucose concentrations were measured using a glucometer (Precision Q.I.D., a gift from Abbott Laboratories, Chicago, IL). Commercially available kits were used for measuring levels of plasma insulin, adiponectin, (Linco Research, St. Charles, MO), non-esterified fatty acids (NEFAs) (Wako Chemicals, Neuss, Germany), and glucose (Sigma Chemical Co., St. Louis, MO) [9, 10].

**Table 1:** **Maternal metabolic phenotype**

	C	HF	C-HF	HF-C
N	10	8	5	8
Maternal Weight at Plug Day (g)	35.9 ±1.8^A^	36.7 ± 1.4^A^	35.4 ± 2.1^A^	35.1 ± 2.9^A^
Maternal Weight at e9.5 (g)	44.3 ± 4.0^A^	39.6 ± 3.1^A^	44.3 ± 1.4^A^	41.7 ± 4.8^A^
Maternal Weight at Sacrifice (g)	62.6 ± 3.6^AB^	51.9 ± 2.8^B^	56.3 ± 1.6^AB^	64.7 ± 6.1^A^
Maternal Weight Gain Throughout the Pregnancy (g)	28.3 ± 1.9^A^	18.2 ± 2.0^B^	20.9 ± 1.4^AB^	24.9 ± 3.7^AB^
Renal Fat (g)	0.6 ± 0.2^A^	0.6 ± 0.2^A^	0.5 ± 0.2^A^	1.3 ± 0.4^A^
Gonadal Fat (g)	1.1 ± 0.4^A^	1.3 ± 0.4^A^	1.4 ± 0.3^A^	1.9 ± 0.6^A^
Visceral Fat (g)	1.7 ± 0.6^A^	1.9 ± 0.6^A^	1.9 ± 0.6^A^	3.3 ± 0.9^A^
Liver (g)	2.3 ± 0.1^A^	2.2 ± 0.1^A^	2.3 ± 0.1^A^	2.5 ± 0.2^A^
Liver/BW	0.036 ± 0.002^A^	0.042 ± 0.002^A^	0.041 ± 0.002^A^	0.039 ± 0.004^A^
Heart (g)	0.20 ± 0.02^A^	0.17 ± 0.02^A^	0.19 ± 0.02^A^	0.21 ± 0.01^A^
Heart/BW	0.0033 ± 0.0002^A^	0.0033 ± 0.0003^A^	0.0035 ± 0.0003^A^	0.0034 ± 0.0003^A^
Adiponectin (ng/ml)	21.9 ± 2.8^AB^	20.6 ± 5.1^AB^	27.4 ± 3.5^A^	12.5 ± 1.8^B^
NEFAs (mg/dl)	2.4 ± 0.2^B^	3.1 ± 0.2^A^	2.3 ± 0.3^AB^	1.7 ± 0.3^B^
Glucose (mg/dl)	127 ± 2^A^	127 ± 5^A^	119 ± 5^A^	134 ± 7^A^
Insulin (pg/ml)	2.3 ± 0.6^A^	1.9 ± 0.1^A^	1.3 ± 0.1^A^	2.0 ± 0.1^A^

Data which are significantly different from each other are denoted by different letters (p < 0.02). All data are presented as the mean ± SEM.

Fetal Study: Pregnant mothers and fetuses were sacrificed at e18.5 of pregnancy, as previously described [9, 10, 23]. Litter number, placental and fetal weights, and crown-rump length (CRL) were recorded, as well as the number of abnormal or dead pups per litter. Fetuses (C: n = 33: HF: n = 53; C-HF: n = 32: HF-C: n = 43) were euthanized by cervical dislocation immediately following dissection from the uterine horn. Fetal plasma was collected and pooled based on diet and genotype. Fetal livers were dissected and frozen in liquid nitrogen and stored at -80°C until analyzed. Genotyping and sex determination of fetuses were performed as previously described [9]. G4+/− and WT fetuses had similar adaptive response to a HF IU, but WT fetuses were the focus of this study because they exhibit the most profound metabolic complications and also to eliminate the potential confounding effects of genetic factors during fetal development [9, 10].

Quantitative Real Time-PCR Analysis: The cDNA of fetal livers was analyzed with qRT PCR for mRNA gene expression (n = 4-6 per diet) as previously described [10, 23]. The RNA was checked for DNA contamination, using PCR with control primers as described previously [29]. Three common housekeeping genes, ubiquitin, b-actin (ACTB), cyclophillin b were used for normalization. For quantitative analysis, all samples were normalized to the genes described above using the ΔCT value method; the control (C) group was used as the reference group. Each sample was measured in triplicate for each gene to assess technical variability [10, 23, 29]. The selection of genes was based on a literature search for genes associated with an altered IU milieu [10]. Sequence-specific primer pairs have been previously provided. Sample size was calculated to detect a 1.5 to 4 fold change in expression level of the gene of interest based on prior findings [10, 23].

Statistical Analysis: Data are presented as the mean ± SEM. Statistical analyses were performed using JMP 7.0 software (SAS Institute, Cary, NC) or Graph Pad Prism software version 5.04 for Windows (Graph Pad Software, San Diego, California). ANOVA was used to test the difference between the means of two (t-test) or more groups and Bonferroni’s post-hoc analysis was performed. When appropriate, the Mann–Whitney two tailed T-test was utilized. A linear regression model was used to determine the effect of sex on fetal BW, CRL and placental weight, and fetal glucose levels [10]. To determine cohort size a standard power calculation was performed based on previous findings [9, 10, 23]. Acceptable study power was agreed a priori to be ≥80% (type-I error of ≤0.20). P < 0.05 was considered statistically significant.

## Results

### HF diet during pregnancy alters maternal weight gain

HF dams gained significantly less weight when compared to C (p < 0.02). C-HF dams tended to gain less weight compared to C, although this did not reach statistical significance (p < 0.07). HF exposure during the first half of pregnancy (HF-C) did not affect maternal weight gain. Maternal liver, heart, renal, visceral fat pad weights (renal and gonadal fat pad) and organ/BW ratios did not differ between the four groups (Table 1).

### HF diet during the first half of pregnancy alters maternal serum adiponectin levels

HF-C dams had decreased adiponectin levels when compared to C-HF (p < 0.02); although there was a trend, this did not reach statistical significance when compared to C dams (p < 0.06). Consumption of a HF diet during pregnancy did not affect insulin and glucose levels in the mothers, but significantly increased serum NEFA levels when compared to C and HF-C (p < 0.01) (Table 1).

### HF diet during the first half of pregnancy reduces litter weight

HF-C dams had significantly smaller litters when compared to the other groups (HF-C vs C p < 0.004; HF-C vs. HF p < 0.02; HF-C vs. C-HF p < 0.02). The number of resorption sites was not significantly different between the four groups, but there was a tendency in the HF-C group to have more resorption sites compared to C and C-HF (HF-C vs. C p = 0.07; HF-C vs. C-HF p = 0.07) (Table 2).

**Table 2:** **Metabolic phenotype of fetuses at e18.5**

	C	HF	C-HF	HF-C
N	33	53	32	43
Total Litter Weight (g)	16.9 ± 2.2^A^	12.9 ± 1.0^AB^	13.9 ± 1.9^AB^	11.6 ± 1.1^B^
Number of Pups per Litter	12.0 ± 1.6^A^	11.0 ± 0.7^A^	11.6 ± 0.3^A^	9.0 ± 0.3^B^
Resorption Sites per Litter	1.8 ± 0.2^A^	2.5 ± 0.3^A^	1.7 ± 0.6^A^	3.5 ± 1.5^A^
Fetal BW (g)	1.40 ± 0.02^A^	1.24 ± 0.01^BC^	1.21 ± 0.02^C^	1.30 ± 0.03^B^
Fetal CRL (cm)	2.55 ± 0.03^A^	2.43 ± 0.03^B^	2.44 ± 0.03^B^	2.43 ± 0.03^B^
Placental Weight (g)	0.107 ± 0.004^A^	0.109 ± 0.004^A^	0.110 ± 0.005^A^	0.111 ± 0.003^A^
Placenta:BW ratio	0.076 ± 0.003^A^	0.089 ± 0.003^B^	0.092 ± 0.004^B^	0.086 ± 0.003^B^
Fetal Glucose (mg/dl)	57.6 ± 3.0^A^	63.2 ± 4.5^A^	82.1 ± 7.8^B^	64.9 ± 5.0^A^

Data which are significantly different from each other are denoted by different letters (p < 0.02). All data are presented as the mean ± SEM.

### HF diet during pregnancy stunts fetal growth without altering placental weight

Exposure to HF at any point during pregnancy was associated with an 8 to 14% decrease in body weight (HF vs. C p < 0.0001; C-HF vs. C p < 0.0001; HF-C vs. C p < 0.02). Of all the HF groups, C-HF had the lowest body weight. HF reduced length (CRL) in all groups (HF vs. C p < 0.004; C-HF vs. C p < 0.02; HF-C vs. C p < 0.004). The decrease was similar in all HF groups compared to the control group (8%). HF exposure did not alter placental weight when compared to the C group. Decreased BW with no corresponding change in placental weight resulted in an increase in placenta:BW ratio in groups exposed to HF at any point during gestation (HF vs C p < 0.004; C-HF vs C p < 0.002; HF-C vs C p < 0.04). No differences in body weight (p = 0.2), placental weight (p = 0.7) or CRL (p = 0.9) was found when the data was analyzed based on fetal sex (Table 2).

### HF diet during the second half of pregnancy results in increased hepatic expression of genes associated with fetal growth

**Figure 2:**
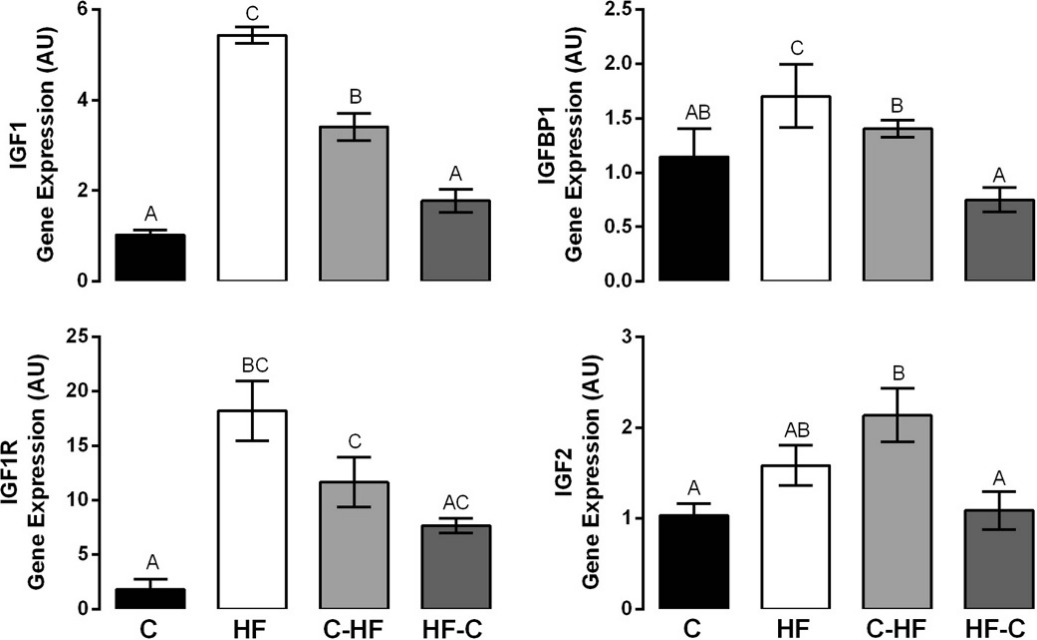
**Relative mRNA Expression of Growth Factors in Fetal Liver at e18.5.** Changes in mRNA expression of genes were determined by qRT-PCR. N = 4-6/diet group. Data points which are significantly different from each other are denoted by different letters (p < 0.01). Bars indicate standard error of the mean. Primer sequences were previously published [10].

Exposure to HF throughout pregnancy increased the hepatic gene expression of insulin-like growth factor 1 (IGF1) (p < 0.0001), insulin-like growth factor 1 receptor (IGF1R) (p < 0.001), and insulin-like growth factor binding protein 1 (IGFBP1) (p < 0.0001), genes involved in fetal growth when compared to the C diet. Hepatic IGF1 and IGFBP1 gene expression was significantly higher in HF fetuses when compared to the C-HF (p < 0.001) and HF-C group (p < 0.0001) (Figure 2).Exposure to a HF diet during the second half of pregnancy (C-HF) significantly increased the hepatic gene expression of IGF1 (p < 0.01), IGF1R (p < 0.01) and insulin-like growth factor 2 (IGF2) (p < 0.01) when compared to the C group. Hepatic IGF1 (p < 0.01), IGF2 (p < 0.01) and IGFBP1 (p < 0.01) gene expression was significantly higher in C-HF fetuses when compared to the HF-C group. Interestingly, although expression of IGF1 was increased in C-HF compared with C group, its expression was decreased compared with HF fetuses (p < 0.05) (Figure 2).The expression of genes involved in fetal growth in HF-C was similar to C fetuses (Figure 2).

### HF diet during the second half of pregnancy increases fetal serum glucose levels and expression of genes associated with hepatic glucose production (HGP)

Control, HF and HF-C fetuses were euglycemic while C-HF fetuses had a significant increase in their glucose levels when compared to all the other groups (p < 0.03). No differences in fetal glucose levels were found when the data was analyzed based on fetal sex (p = 0.8) (Table 2).

**Figure 3:**
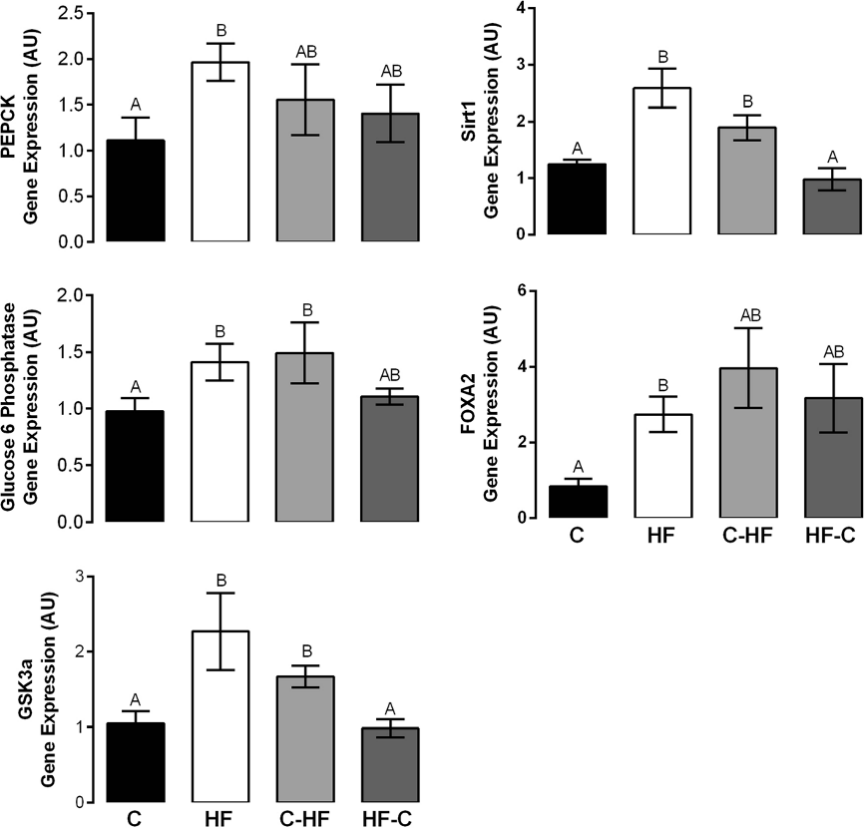
**Relative mRNA Expression of Glucose Production Genes in Fetal Liver at e18.5.** Changes in mRNA expression of genes were determined by qRT-PCR. N = 4-6/diet group. Data points which are significantly different from each other are denoted by different letters (p < 0.04). Bars indicate standard error of the mean. Primer sequences were previously published [10].

Exposure to a HF diet throughout pregnancy (HF) significantly increased the expression of hepatic genes associated with glucose metabolism such as phosphoenolpyruvate carboxylase (PEPCK) (p < 0.04) and glucose 6-phosphatase (G6Pase) (p < 0.01), rate-limiting enzymes of GNG; glycogen synthase kinase alpha (GSK3a) (p < 0.01), a rate-limiting enzyme of glycogenolysis; NAD^+^ −dependent protein deacetylase (SIRT1) (p < 0.002), a PGC1α activator that regulates expression of GNG genes; and Forkhead Box Protein A2 (FOXA2) (p < 0.003), a transcription factor that regulates the expression of genes such as PEPCK when compared to a C diet. Hepatic GSK3a (p < 0.01) and SIRT1 (p < 0.01) gene expression were significantly elevated in the HF fetuses when compared to HF-C group (Figure 3).Exposure to a HF diet during the second half of pregnancy (C-HF) significantly increased the hepatic gene expression of G6Pase (p < 0.01), GSK3a (p < 0.01), and SIRT1 (p < 0.01) when compared to a C diet. Hepatic GSK3a (p < 0.01) and SIRT1 (p < 0.01) gene expression were significantly elevated in the C-HF fetuses when compared to HF-C group (Figure 3).

The pattern of expression of genes involved in glucose metabolism in HF-C fetuses was similar to C fetuses.

Gene expression of glucose transporter 2 (GLUT2), glycogen synthase kinase beta (GSK3b) and insulin receptor (INSR) which encode proteins that participate in hepatic glucose metabolism were not significantly affected by the diet (Table 3).

**Table 3:** **List of genes that were not differentially expressed in fetal liver at e18.5**

Gene name	Gene symbol	Gene sequence (Forward and Reverse)	p value
Glycogen-synthase kinase b	GSK3b	CGGGACCCAAATGTCAAACT	NS
		TCCGAGCATGTGGAGGGATA	
Glucose transporter 2	GLUT2	GTGTGCAGCAGCCTGTGT	NS
		CAGTGAAGGCCGTGTTGAC	
Insulin Receptor	INSR	CCTGAAAAGTCACCTCCGTTCT	NS
		TTCAAGTATGCCATGCCATCA	
Hydroxysteroid (11-beta) dehydrogenase 1	HSD11B1	GGGAAATGACCCAGCCTATG	NS
		CGTGGAAAAGAACCCATCCA	
Hydroxysteroid (11-beta) dehydrogenase 2	HSD11B2	CGGGCAGTTCCTGAATTCAC	NS
		GCATCGATGATGGCATCTACA	
Sterol regulatory element binding transcription factor 1	SREBF1	CCAGAGGGTGAGCCTGACAA	NS
		AGCCTCTGCAATTTCCAGATCT	
Sterol regulatory element binding transcription factor 2	SREBF2	CACCAGCTGCACATCACAG	NS
		ACTCGGCCAGGTTCACAG	

N = 4-6/diet group; NS = not significant.

### HF diet during the second half of pregnancy results in increased hepatic expression of genes associated with oxidative stress and inflammation in the fetus

**Figure 4:**
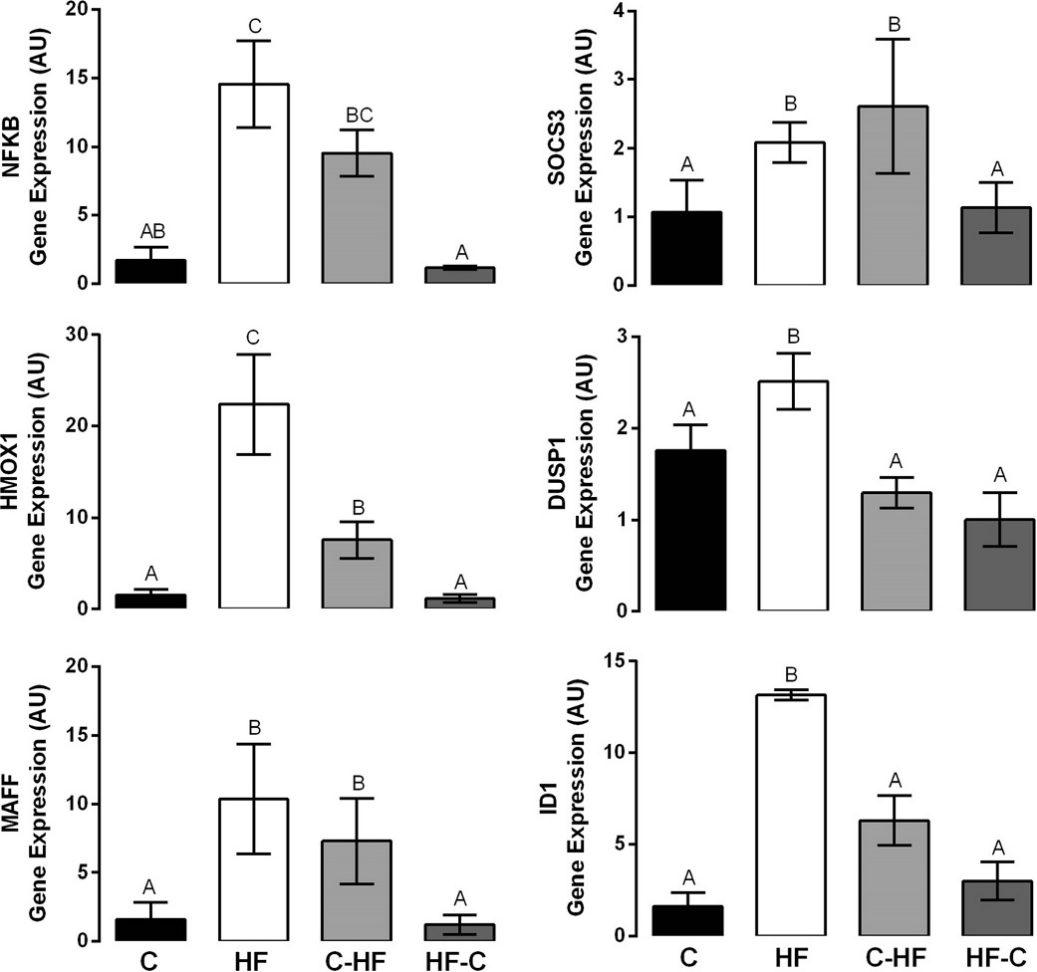
**Relative mRNA Expression of Genes associated with Oxidative Stress in Fetal Liver at e18.5.** Changes in mRNA expression of genes were determined by qRT-PCR. N = 4-6/diet group; Data points which are significantly different from each other are denoted by different letters (p < 0.01). Bars indicate standard error of the mean. Primer sequences were previously published [10].

Exposure to a HF diet throughout pregnancy (HF) significantly increased the expression of hepatic genes associated with inflammation such as nuclear factor kappa B 1 (NFKB1) (p < 0.001), heme oxygenase 1 (HMOX1) (p < 0.001), musculoaponeurotic fibrosarcoma oncogene homolog F (MAFF) (p < 0.001), cytokine signaling protein 3 (SOCS3) (p < 0.01), dual specificity protein phosphatase 1 (DUSP1) (p < 0.01), and inhibitor of DNA binding 1 (ID1) (p = 0.0001) when compared to a C and HF-C diet. Hepatic HMOX1 (p < 0.001), DUSP1 (p < 0.01) and ID1 (p < 0.01) gene expression were significantly elevated in the HF fetuses when compared to C-HF group (Figure 4).Exposure to a HF diet during the second half of pregnancy (C-HF) significantly increased the hepatic gene expression of HMOX1 (p < 0.01), MAFF (p < 0.01) and SOCS3 (p < 0.01) when compared to C and HF-C groups. Similarly, expression of NFKB1 was increased in C-HF fetuses when compared to HF-C (p < 0.01) (Figure 4).The pattern of expression of genes involved in inflammation in HF-C fetuses was similar to the C group (Figure 4).

### HF diet during pregnancy does not alter hepatic expression of genes associated with cortisol or lipid metabolism in fetal liver

Exposure to HF at any point during pregnancy did not affect expression of genes encoding hepatic enzymes that catalyze cortisol metabolism such as hydroxysteroid (11-beta) dehydrogenase 1 (HSD11B1) and hydroxysteroid (11-beta) dehydrogenase 2 (HSD11B2). Hepatic expression of sterol regulatory element binding transcription factors 1 and 2 (SREBF1 and SREBF2), transcriptional activators required for lipid homeostasis, were not significantly affected by exposure to HF at any point during pregnancy (Table 3).

## Discussion

Prenatal exposure to a HF diet is associated with increased risk for MetS. Using a previously characterized mouse model [9, 10, 22, 23], we sought to determine whether the timing of exposure to maternal HF diet affected fetal growth and expression of hepatic genes known to influence fetal growth and postnatal insulin resistance and relate it to possible changes in maternal glucose metabolism.

The timing of exposure to maternal HF resulted in different outcomes with respect to litter size, fetal body weight, crown rump length, and hepatic gene expression. These changes were accompanied by differences in maternal weight gain and adiponectin levels.

Consistent with our previous findings the number of fetuses was not altered in the group exposed to HF throughout pregnancy [9, 10]. However, a decreased number of fetuses were observed in HF-C dams compared to all other groups. Decreased number of fetuses was accompanied with an increase in the number of resorption sites. These findings suggest that the period of transition from HF to control diet is a critical period in sustaining embryo survival.

While reduced fetal/birth weight in response to HF IU has been reported in some studies [21, 30, 31], other studies have reported no change [32, 33] or increased fetal/birth weight [34, 35]. Some reasons for these discrepancies include: duration of HF exposure; presence/absence of maternal obesity; diet composition; and species studied. In this animal model, the dams exposed to a HF diet at any point during their pregnancy or periconception are not obese. When compared to C group HF dams had a significantly reduced body weight gain.

Despite no significant reduction in maternal weight gain, fetuses exposed to HF in the second half of pregnancy (C-HF) had the lowest body weight at e18.5, followed by fetuses exposed to HF throughout pregnancy (HF). Interestingly, similarly to the BW gain seen in HF-C dams, fetuses in which the diet was switched from HF to C during the second half of pregnancy had an increase in BW compared to HF and C-HF groups. These data suggests that similar to humans, late pregnancy in mice is also the critical period for the most rapid fetal growth [36]. The data also suggests that maternal weight gain may not reflect fetal growth.

CRL was significantly reduced in all HF groups. These data indicate that although the timing of exposure to HF during pregnancy influences fetal body weight it is not enough to prevent fetal growth restriction as indicated by CRL measurements.

Placental weight was not affected by HF diet at any point during pregnancy. The placenta is a highly efficient organ that integrates signals from the mother and fetus and serves to match fetal growth demands [16, 37]. The phenomenon of compensatory placental expansion in infants with low birth weight has been well documented; however, placental function may not necessarily correlate with placental weight [38]. HF exposure did not alter placental weight but did decrease fetal body weight, thus the increase in the placental weight: BW ratio observed in the HF groups compared to the control group may reflect a placental compensatory response to improve nutrient transfer. Studies have demonstrated that elevated placental IGFBP-1 is associated with an increase in the size of the labyrinth zone, the area of nutrient exchange in the mouse placenta and an increased placental weight: BW ratio [39]. Future studies are needed to confirm this hypothesis.

The greatest changes in maternal weight gain and gene expression were seen in HF dams and their fetuses suggesting that increased duration of HF exposure elicits a greater effect. Consumption of a HF diet during pregnancy did not affect maternal glucose or insulin levels at e18.5. However, in addition to having smaller litters, HF-C dams had decreased adiponectin levels when compared to C-HF dams. In contrast, C, and HF dams had similar adiponectin levels and litter size. Adiponectin is secreted from both adipose tissue and placenta [40]. It is inversely correlated with body fat percentage in adults and regulates glucose and fatty acid oxidation [41]. Increased levels are generally associated with insulin sensitivity. There have been inconsistent reports regarding adiponectin levels during pregnancy [42, 43]. Decreased adiponectin levels have been seen in gestational diabetes mellitus while elevated adiponectin levels have been observed with preeclampsia [42, 44], suggesting that adiponectin levels are altered during pathological gestations. Adiponectin can be found in cord blood [45]; therefore it is plausible that decreased adiponectin levels seen in HF-C dams could be a link between the altered maternal metabolic environment and poor pregnancy outcomes seen in this group. At this point, it is not clear why adiponectin levels were not different among the other groups.

IGFs and IGFBPs are thought to play an important role in fetal growth [46]. Liver is the predominant source of circulating IGFs. IGF2 is the primary growth factor during embryonic growth, and IGF1 is the dominant growth regulator during late gestation [47]. Reduced IGF1 action, due to polymorphisms or heterozygous mutations in IGF1 or the IGF1 receptor (IGF1R), leads to low birth weight [48]. In humans, IGF1 and IGF2 serum levels are significantly reduced in SGA fetuses [49]. HF and C-HF fetuses were growth restricted despite having increased IGFs and IGF1R gene expression. A previous study in nutrient restricted ewes demonstrated increased IGF1 and IGF2 receptors in adipose tissue despite growth restriction [50]. This may indicate reduced IGF action arising from decreased IGF bioavailability (mRNA expression that may not reflect serum IGF concentration) and/or bioactivity (IGF resistance or altered IGF signaling).

Interestingly, although expression of IGF1 was increased in C-HF compared with C, its expression was decreased when compared with HF. This is important to note because C-HF fetuses have the lowest body weight. Thus, it is possible that changes in IGF1 expression in HF fetuses are able to compensate for the differences in BW seen at e18.5 in both groups, and the lack of increased in the IGF-1 expression C-HF may, in part, explain reduced BW in this group.

Altered IGF1 signaling has been seen in human placentas from pregnancies with growth restricted fetuses [51]. These placentas may be viewed as IGF-resistant tissue where IGF1 signaling is blunted due to reduced expression of the IGF1R; impairment of the IRS-2/phosphatidyl inositol 3-kinase pathway; and reduced p38 and c-Jun N-terminal kinase activation [51]. In addition, IGFBP-1 mRNA expression, which was also elevated in our model and other models of growth restriction [39], could act as a potent inhibitor of IGFs interacting with the IGF1R.

Glucose levels were significantly increased in C-HF fetuses. These changes were accompanied by an increase in hepatic IGF2 expression. Increased IGF2 expression in the fetal liver has been associated with fetal undernutrition [52] and neonatal diabetes [53], and possible HF feeding during pregnancy [54]. Increased IGF2 mRNA levels have been associated with impaired placental nutrient transport [55], suggesting that the elevated glucose levels seen in the C-HF model may represent an increase in fetal hepatic glucose production as an adaptive response to a reduced placental nutrient transport.

The principal cause of fasting hyperglycemia in T2DM, a disease burden for SGA babies, is hepatic insulin resistance leading to unsuppressed GNG [18]. Although changes in hepatic GNG may not be directly inferred from gene expression data [56], the observed alterations in the gene expression, (PEPCK, G6P and GSK3a) seen with C-HF are consistent with increased hepatic glucose production and may, in part, explain the observed increase in fetal glucose levels. In addition, HF exposure increased hepatic SIRT1 expression. SIRT1 is an important regulator of hepatic GNG [57], and increased expression of SIRT1 may contribute to the increased glucose levels observed in C-HF fetuses.

Activation of glucose production in response to HF may be an adaptive response to maternal malnutrition or impaired placental nutrient transport or may represent hepatic insulin resistance. Insulin is the dominant mechanism for suppressing GNG gene expression and glucose production [58]. We previously demonstrated that HF exposure does not alter insulin levels under basal conditions [10]. Although we did not investigate the phosphorylation status of proteins downstream of the insulin receptor such as V-akt murine thymoma viral oncogene homolog 1 (Akt) or GSK3, mRNA expression of GLUT2 and the insulin receptor, two important components of the insulin signaling pathway, were not altered suggesting normal hepatic insulin action. Thus, it is possible that the increased glucose levels seen in the C-HF model may not reflect hepatic insulin resistance [59] but reflect either increased hepatic glucose production or decreased peripheral glucose disposal. Mechanistic studies in isolated fetal hepatocytes are needed to test these hypotheses. In contrast to the C-HF fetuses, HF fetuses have normal glucose levels. Thus, it is plausible that the elevated glucose levels seen in C-HF when compared to the HF fetuses were a response to the acute change in the diet.

Inflammatory cytokines are a proposed link between obesity, insulin resistance and metabolic disease [60]. Studies in non-human primates [21] and mice [10] exposed to a maternal HF diet show that fetuses are SGA and develop an inflammatory-oxidative stress response and liver lipotoxicity compared to controls exposed to a normal diet. The expression of genes associated with cellular stress and inflammation (MAFF, HMOX1, DUSP1, SOCS3, ID1) in the HF and C-HF fetal liver are consistent with these studies and suggest a mechanism where exposure to HF, either throughout pregnancy or during the second half of pregnancy, increases the risk of MetS in later life [10, 21].

The expression of key enzymes and transcription factors involved in insulin signaling is turned on during the second half of pregnancy [28]. Therefore, late gestation may represent a critical window during hepatic development when exposure to HF may have a profound impact on the future metabolism of the offspring [61]. Although many of the changes observed by exposure to HF in the second half of pregnancy were similar to those observed in fetuses exposed to HF throughout pregnancy, future studies to clarify the long-term implications of the different exposures are warranted.

One question that still remains is whether HF exposure during the second half of pregnancy is enough to program MetS in the offspring or if the offspring needs to be continuously exposed to HF throughout pregnancy and lactation. Although the current studies do not answer this question, our previous findings have demonstrated that some histone modifications that occur in fetal livers in response to HF exposure persist up to 5 weeks of age [24].

## Conclusions

In conclusion, exposure to HF diet during the first half of pregnancy determines litter size while exposure to HF during the second half of pregnancy leads to dysregulation of expression of key genes responsible for fetal growth, hepatic glucose production and oxidative stress; some of these changes seem to be exacerbated by the length of the HF exposure. Whether these alterations contribute to the increased susceptibility of MetS in adulthood remains to be determined, but our findings suggest that dietary modifications made during pregnancy can prevent alterations in hepatic gene expression that are associated with MetS.
